# Multi-Species Comparative Analysis of the Equine ACE Gene Identifies a Highly Conserved Potential Transcription Factor Binding Site in Intron 16

**DOI:** 10.1371/journal.pone.0055434

**Published:** 2013-02-08

**Authors:** Natasha A. Hamilton, Imke Tammen, Herman W. Raadsma

**Affiliations:** 1 ReproGen-Animal Bioscience Group, Faculty of Veterinary Science, University of Sydney, Camperdown, New South Wales, Australia; 2 ReproGen-Animal Bioscience Group, Faculty of Veterinary Science, University of Sydney Camden, New South Wales, Australia; Ecole Normale Supérieure de Lyon, France

## Abstract

Angiotensin converting enzyme (ACE) is essential for control of blood pressure. The human ACE gene contains an intronic *Alu* indel (I/D) polymorphism that has been associated with variation in serum enzyme levels, although the functional mechanism has not been identified. The polymorphism has also been associated with cardiovascular disease, type II diabetes, renal disease and elite athleticism. We have characterized the *ACE* gene in horses of breeds selected for differing physical abilities. The equine gene has a similar structure to that of all known mammalian *ACE* genes. Nine common single nucleotide polymorphisms (SNPs) discovered in pooled DNA were found to be inherited in nine haplotypes. Three of these SNPs were located in intron 16, homologous to that containing the *Alu* polymorphism in the human. A highly conserved 18 bp sequence, also within that intron, was identified as being a potential binding site for the transcription factors Oct-1, HFH-1 and HNF-3β, and lies within a larger area of higher than normal homology. This putative regulatory element may contribute to regulation of the documented inter-individual variation in human circulating enzyme levels, for which a functional mechanism is yet to be defined. Two equine SNPs occurred within the conserved area in intron 16, although neither of them disrupted the putative binding site. We propose a possible regulatory mechanism of the *ACE* gene in mammalian species which was previously unknown. This advance will allow further analysis leading to a better understanding of the mechanisms underpinning the associations seen between the human *Alu* polymorphism and enzyme levels, cardiovascular disease states and elite athleticism.

## Introduction

Angiotensin converting enzyme (ACE) is an essential component of the renin-angiotensin system and plays an important role in the control of blood pressure, renal function and male fertility [1]. The presence of a 287 bp *Alu* insertion/deletion (I/D) polymorphism with a high minor allele frequency (0.4–0.47) within the *ACE* gene [2], [3], combined with the intrinsic function of the enzyme, has resulted in over 500 association studies between the human *ACE* gene and a wide range of disorders, most notably cardiovascular disease, type II diabetes and related renal disease [4]–[7]. More than 20 studies have explored a possible association with extreme athleticism, with conflicting results. In many studies, the insertion (I) allele has been associated with anabolic response to training and elite endurance performance, whilst the deletion (D) allele was associated with sprint or power performance [8]–[12], and both variants have been associated with response to strength training [13], [14]. However, some studies have found no connection with athletic performance [15]–[18].

The I/D polymorphism, which is found in intron 16, appears to account for 28–47% of the inter-individual variation in serum ACE levels, either due to increased expression of the D allele mRNA, or lower stability of the I allele mRNA [3], [19], [20]. Circulating enzyme levels were found to be influenced by the action of a major codominant gene, with adults homozygous for the I allele having significantly lower circulating enzyme levels than homozygotes for the D allele. Heterozygotes fall in between these levels [3], [19]. The suggestion that an unidentified intronic silencer element is eliminated by the deletion variant, and thus increases observed circulating enzyme levels, has been ruled out [21]. Other studies have hypothesised that other polymorphisms are responsible for the effects attributed to the I/D, and instead indicate that potentially two functional variants exist, probably in the 3′ region of the gene, accounting for these effects [22]–[24]. However, these studies were unable to identify the functional variant(s) and only investigated a selection of known ACE polymorphisms. Furthermore, the mode of action of alternate allelic forms of the *ACE* gene on variation in circulating enzyme levels in addition to performance is yet to be elucidated.

The human *ACE* gene spans 21308 bp [GenBank:NG_011648] of which 4422 bp comprises coding sequence across 26 exons [25]. It encodes two commonly expressed isozymes, the larger of which is membrane bound and primarily found in both epithelial and endothelial cells. In particular, vascular cells from the brain and lung produce large amounts of ACE, as do the brush border cells of kidney tubules, while all mammalian endothelial cells appear to produce ACE (endothelial or somatic ACE, sACE) [26]. Vascular endothelial cells also release a circulating form of the enzyme by cleaving it from the membrane bound tail [27], [28]. This large isozyme is transcribed from exons 1 to 26, excluding exon 13. The smaller ACE variant is only expressed after puberty in the germinal cells of the testes, is encoded by exons 13 to 26 through initiation of a separate promoter in intron 12, and is known as testicular ACE (tACE). A feature of the *ACE* gene is a high degree of homology between two distinct regions of the gene, namely exons 4 to 11 (region 1) and 17 to 24 (region 2). The exons in regions 1 and 2 have conserved codon phases, are up to 80% similar, are identical lengths, and are likely the result of a gene duplication event that occurred before mammalian radiation [29].

The horse presents itself a suitable species to study the *ACE* gene in a biological model of athletic performance. Horses are unique in livestock breeding in that they have almost exclusively been selected for athletic performance, ranging from extreme speed, endurance, or heavy draught performance. Additionally, horses have been shown to exhibit similar interanimal variation in circulating ACE levels as observed in humans [30]. We present here a comprehensive characterization of the equine angiotensin converting enzyme gene in comparison with other mammalian *ACE* genes, in particular the human, to shed light on the possible mode of action of *ACE* gene polymorphisms on circulating enzyme levels and thus performance in an athletic animal model.

## Materials and Methods

### Ethics Statement

All horses sampled for this project were covered by Animal Ethics protocols N02/5-99/1/2946 and N00/3-2002/1/3535, as approved by the University of Sydney Animal Ethics Committee.

### Animals

Horses of seven different breeds including racing Thoroughbreds (TB), Standardbreds (SB), Draught/Heavy Horses (HH) (Shires and Clydesdales), endurance Arabians (AR), Quarter Horses (QH) and ponies (PO) were included in this study. The TBs were selected based on minimal degree of relatedness according to pedigree; the ARs were selected for successful endurance performance at distances over 80 km; and for all other breed samples animals were selected at random. Blood samples were collected by venipuncture of the jugular vein for extraction of DNA.

### DNA and RNA Extraction

DNA was extracted using a QIAGEN Plasmid Midi Kit (Qiagen, Hilden, Germany) from BAC clone 801F9, which was identified as containing the equine *ACE* gene. This clone was supplied from the INRA horse BAC library by Dr. Francois Piumi, INRA [31]. Equine DNA was extracted from fresh blood as previously described [32] or as per manufacturer’s instructions from frozen blood samples using a QIAamp® DNA Blood Mini Kit (Qiagen). RNA was extracted from blood using an RNeasy® Mini Kit (Qiagen).

The DNA samples were combined to create 3 pools, composed of TB (n = 10), AR (n = 14), and mixed breeds (MB) (including 2 each of HH, SB, TB, QH and PO) for the discovery of polymorphisms. Common polymorphisms discovered in the pools were also typed across a panel of 40 horses (10 each of TB, AR, SB and HH) to obtain allele frequency and haplotype information.

### Primers

Since the equine *ACE* coding sequence was not available at the commencement of this study, primers were selected based on the aligned cDNA sequences of the human, rabbit, rat and chicken genes [GenBank:J04144, X62551, AF201332 and L40175]. One of three standard M13 tail sequences was synthesized on the 5′ end of the primers (as listed in Table S1), to allow incorporation of a fluorescent label into the PCR product [33].

### PCR and Sequencing

Reverse transcriptase reaction PCR was carried out with 2 µg total RNA and reagents from Promega (Madison, WI, USA) and Life Technologies (Grand Island, NY, USA). The cDNA was diluted to a 1 in 5 dilution and 1 µL used as a template in PCR with a total volume of 25 µL.

PCR reactions for genomic, BAC and cDNA were performed in 25 µL volumes containing 20 ng of purified DNA and reagents from Fisher Biotech (Perth, Australia). Primer sequences for determining the gene sequence and screening the DNA pools are shown in Table S1.

PCR product was cleaned up using ExoSapIT (GE Healthcare Life Sciences, Buckinghamshire, England) or JetQuick PCR Purification Spin columns (Genomed, Löhne, Germany) as per manufacturer’s instructions. Sequencing was performed using Sequitherm Excel II sequencing kits (Epicenter, Madison, WI, USA) and IRD-labeled M13 primers (MWG Biotech, Ebersberg, Germany) on a LiCOR 4200 automated sequencer (Lincoln NE, USA), or by using Big-Dye Terminators (BDT) version 3.1 (Applied Biosystems, CA, USA) on an ABI PRISM 3100 Genetic Analyser (Applied Biosystems). Direct BAC sequencing was used to develop the sequence at the 5′ and 3′ ends of the gene and within intron 14 as previously described [34].

### Polymorphism Identification and Genotyping

Polymorphisms were identified by comparing the chromatograms of pooled sequence with that of a single animal, and confirmed by genotyping of animals within the pool. Restriction fragment length polymorphism (RFLP) or sequencing (when no restriction enzymes were available) was used to genotype individuals (Table 1).

**Table 1:** Genotyping conditions for nine common equine *ACE* gene polymorphisms.

SNP	Position	Variant	Breeds	Genotyping Method
1	Intron 5	c.868+25A>G	AR, TB	Eliminates *NspI* restriction site
2	Intron 8	c.1363+146G>T	AR, TB	Introduces *BamHI* restriction site
3, 4	Intron 16	c.2326+89C>G; c.2326+178G>A	AR, TB; AR, TB, SB, HH	Sequencing
5	Intron 16	c.2326–583G>T	AR, TB, SB, HH	Sequencing
6, 7	Intron 20	c.2933+58G>A>C; c.2933+115G>T	AR, TB; AR, TB	Sequencing
8	Intron 21	c.3157+39C>A	TB, AR, HH, BAC	Sequencing
9	Exon 26	p.Arg1290His	HH	Eliminates *AciI* restriction site

The breeds column indicates the breeds the polymorphism was indentified in this study. Variant annotation was according to the guidelines of the Human Genome Variation Society. Primer sequences are listed in Table S1.

### Bioinformatics and Statistical Analyses

Sequencher (Gene Codes, MI USA) was used to visualise chromatograms. We determined equine *ACE* haplotypes to allow for association testing in the future using Phase 2.0.2 [35] as we lacked parent-progeny combinations to derive haplotypes empirically. Cross species amino acid similarity and identity was scored using the program MatGAT [36]. Hydropathy analysis of predicted amino-acid sequence was carried out using the Kyte and Doolittle scale with a window size of 11 residues [37]. The transmembrane domain was identified using the Statistical Analysis of Protein Sequences (SAPS) package [38] and SignalP used to predict signal peptide cleavage sites [39]. Orthologs of the equine ACE gene were identified with Ensembl and used to create conservation plots with VISTA tools, using the LAGAN global alignment [40]. Multiple sequence alignments were performed using ClustalW2 with default settings. Phylogenetic analysis was performed with PhyML using the default settings on the horse, dog, cat, dolphin, cow, rabbit, elephant, human, chimpanzee, rat and mouse orthologous *ACE* genes [EnSembl: ENSECAG00000012910, ENSCAFG00000012998, ENSFCAG00000002078, ENSTTRG00000001667, ENSBTAG00000024950, ENSOCUT00000001559, ENSLAFG00000006295, ENST00000290866, ENSPTRT00000049041, ENSRNOT00000010627 and ENSMUST00000001963 respectively], using the elephant as an outgroup [41]. The internet based tools ConSite, TFSearch, MatInspector, Alibaba2, MAPPER and rVISTA were all used to identify transcription factor binding sites [40], [42]–[47].

## Results and Discussion

### Equine ACE Gene and Predicted Amino Acid Sequence

**Figure 1:**
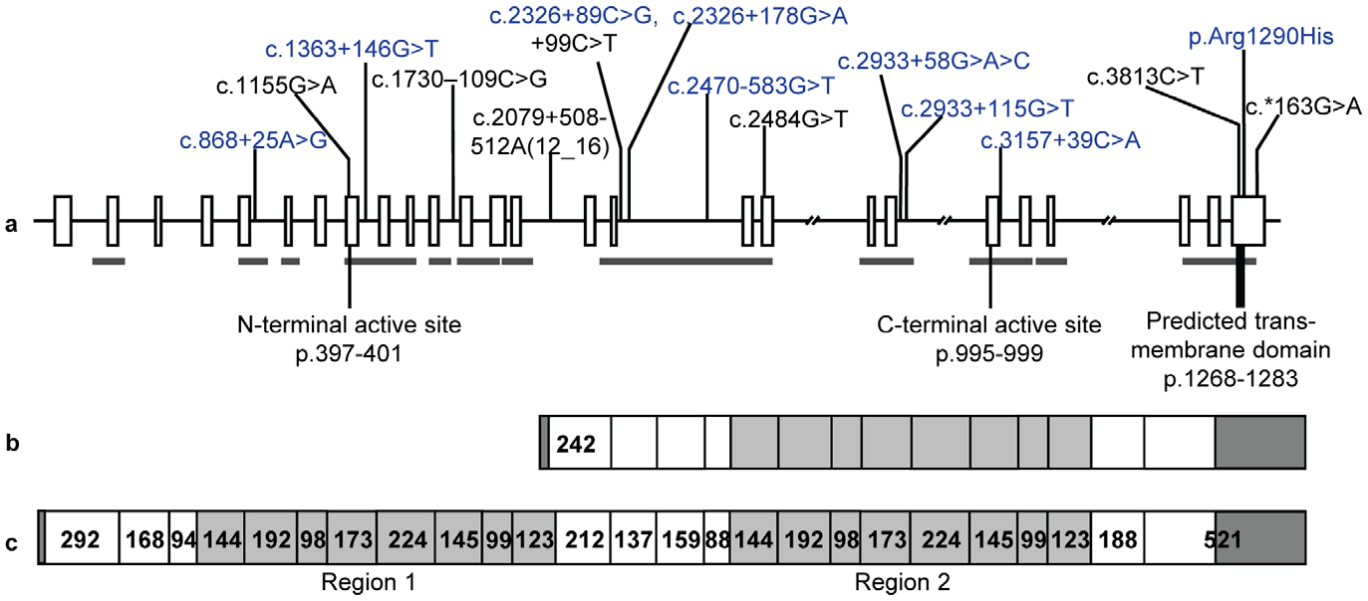
Schematic representation of the equine *ACE* gene structure. The genomic structure (a) depicts exons (boxes), introns (bars) and the locations of polymorphisms identified in this study. The broken bars indicate where sequencing was not completed through introns. The grey bar beneath the gene indicates areas screened in the pools for polymorphisms. Common polymorphisms used for haplotype analysis are indicated in blue font, and the active sites and transmembrane domain are indicated. The predicted testicular (b) and endothelial (c) transcripts, including exon sizes (bp), are shown. The duplicated areas of the gene (regions 1 and 2) are shaded in light grey and the predicted UTR’s are shaded dark grey.

The genomic sequence of the equine *ACE* gene [GenBank:JX227848] was derived with the exception of the central regions of 3 large introns (18, 20 and 23), which comprise 3.54% of the total predicted gene sequence when compared to the horse genome reference sequence (Figure 1). In agreement with the structure of other mammalian *ACE* genes, the equine gene consisted of 26 exons. The cDNA showed a high level of conservation, both in sequence and exon size, between the horse and rabbit, human, rat and mouse genes, which were 87, 86, 84 and 84% homologous respectively. Sequencing the cDNA confirmed that exon 13 is not transcribed in the somatic form of equine ACE (sourced from leukocytes). The exons ranged from 75% (exon 26) to 95% (exon 9) similarity between horse and human (Table 2), with only exons 1, 13 and 26 differing in size from the human gene. The additional nucleotides in exons 1 and 13 in the horse compared to the human form part of the signal peptides and are thus cleaved from the mature enzymes. Similarly, the nucleotides increasing the size of exon 26 in the horse compared to the human are found in the 3′ untranslated region (UTR). A high degree of homology was observed between exons 4 to 11 (region 1, Figure 1) and 17 to 24 (region 2, Figure 1), consistent with evidence for ancestral duplication of this gene [25]. In contrast to the exons, the introns showed little conservation across species in sequence, although the sizes were roughly similar, with the exceptions of intron 12 and 16. Intron 12 was 81% homologous to human intron 12, allowing identification of the putative testicular ACE promoter elements, while the first half of intron 16 was up to 77% similar to the homologous human intron. Intron 14 was found to contain an equine repetitive element-2 (ERE-2) [48].

**Table 2:** Comparison of the human and horse *ACE* exons and intron sequences.

Number	Exon Size (bp)			Intron Size (bp)	
	ECA	HSA	% Identity	ECA	HSA
1	*292*	*271*	81.5	616	587
2	168	168	85.1	612	908
3	94	94	93.6	686	668
4	144	144	87.5	464	424
5	192	192	89.1	577	562
6	98	98	89.8	399	377
7	173	173	86.1	331	727
8	224	224	85.3	345	339
9	145	145	95.2	302	286
10	99	99	92.9	290	290
11	123	123	93.5	364	358
12	212	212	85.8	291	299
13	*242*	*228*	77.7	126	169
14	137	137	92.7	1083	1184
15	159	159	84.3	278	270
16	88	88	83.0	2095	1574^a^
17	144	144	88.9	149	149
18	192	192	90.1	>1584	1821
19	98	98	91.8	201	157
20	173	173	87.9	>1513	2054
21	224	224	87.5	311	262
22	145	145	86.9	334	305
23	99	99	91.9	>2105	1923
24	123	123	93.5	360	281
25	188	188	87.8	175	151
26	*521*	*481*	75.0		

Exons and introns are compared by size, and exons by sequence identity. Cross species identity between the introns was generally too low to align and thus calculate. Introns 18, 20 and 23 were not fully sequenced. Exons shown in italics differ in size between the two species.

a Human intron 16 varies in length from 1574–1859 bp, depending on the presence or absence of the I/D polymorphism.

The first 360 bp of the 5′ region upstream of exon 1 was sequenced. This included some of the equine sACE promoter region, which was aligned with the human [GenBank:AF118569.1] and mouse [GenBank:M34433] promoter sequences (Figure S1, part a). Only the 130 bp directly upstream of the putative transcriptional start site (TSS) showed a significant homology of 75% and 88% to the human and mouse promoters respectively. This sequence corresponds to the 132 bp known to confer promoter activity to the human gene, and includes three potential SP1 binding sites homologous to the equivalent functional sites in the human [49]. No consensus CCAAT element was identified, which is in agreement with the comparison of human and mouse genes [25], [50]. A similar comparison was performed for the tACE promoter (Figure S1, part b). The testicular promoter TTATT sequence was 15 bp upstream of the predicted TSS while the cAMP-responsive element binding site was conserved between the horse, human and mouse sequences [51].

The equine somatic ACE sequence including start and stop codons comprised 3942 nucleotides (1313 amino acids). The testicular enzyme comprises 737 amino acids, 72 of which are unique to this particular isozyme. The aa sequence showed highest similarity to the cow and the least to the rat (Table 3). The signal peptide cleavage sites were predicted to be between residues Ala36 and Leu37 in sACE and Ser28 and Gln29 in tACE, making the mature enzymes 1277 and 709 aa long. Two metalloprotease active sites were predicted to be present in exons 8 and 21. The first showed the consensus sequence (H-E-M-G-H) present in other species. The second site differs by one residue, containing an isoleucine instead of a methionine in the central position, although the significance (if any) of this is unknown. Hydropathy analysis indicated that the C-terminal segment most probably anchors the enzyme to the cell membrane, similar to the pig [52], and amino acids 1268–1283 were identified as the most likely transmembrane segment.

**Table 3:** Homology of equine ACE amino acid sequence to 5 different species.

Species	Genbank Accession No.	Identity (%)	Similarity (%)
Cow (*Bos Taurus*)	1919242A	87.1	93.4
Human (*Homo sapiens*)	NP_000780	86.7	93.5
Chimpanzee (*Pan troglodytes*)	AAG31358	86.5	93.2
Mouse (*Mus musculus*)	P09470	83.2	92.5
Rat (*Rattus norvegicus*)	AAP80808	82.9	92.1

**Figure 2:**
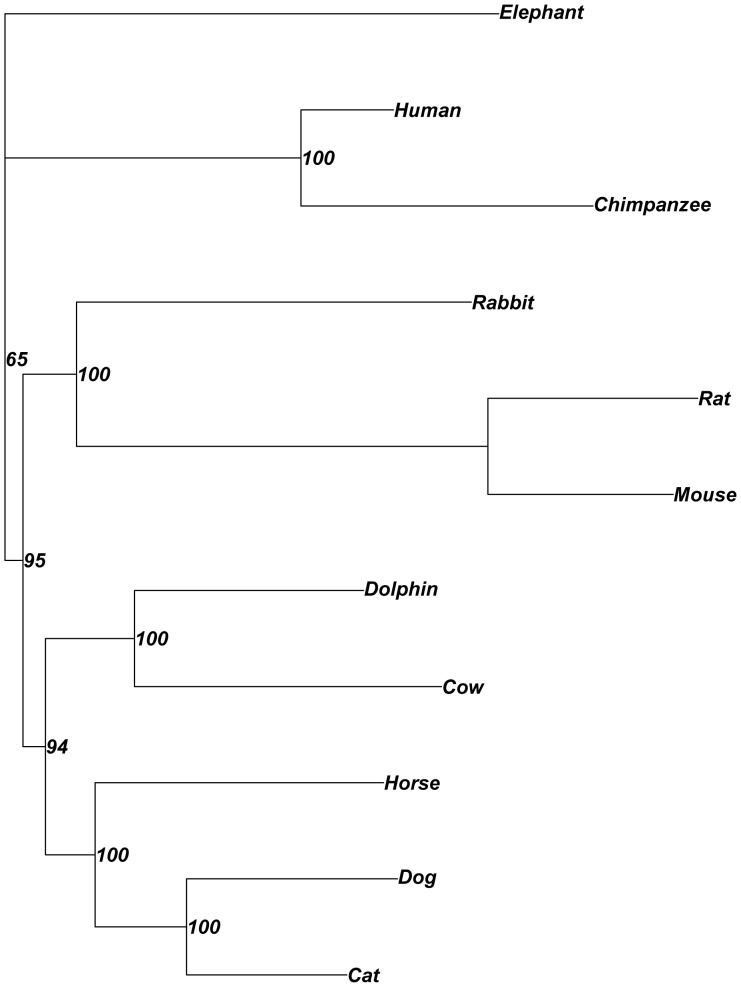
Maximum likelihood consensus tree of ACE cDNA sequence across 11 species. The horse, dog, cat, dolphin, cow, rabbit, elephant, human, chimpanzee, rat and mouse orthologous *ACE* genes were included, with the elephant designated as the outgroup [EnSembl: ENSECAG00000012910, ENSCAFG00000012998, ENSFCAG00000002078, ENSTTRG00000001667, ENSBTAG00000024950, ENSOCUT00000001559, ENSLAFG00000006295, ENST00000290866, ENSPTRT00000049041, ENSRNOT00000010627 and ENSMUST00000001963 respectively]. The GTR model of nucleotide substitution was applied and bootstrap branch supports are shown (100). Transition/transversion ratio, number of invariable sites and gamma distribution parameters were estimated from the data.

Phylogenetic analysis of the cDNA was carried out on the horse, dog, cat, dolphin, cow, rabbit, elephant, human, chimpanzee, rat and mouse orthologous *ACE* genes. The resultant ML consensus tree (Figure 2) showed good agreement with the current accepted phylogenetic relationships of placental mammals, although bootstrapping indicated lower confidence in the position of the primates and outgroup Proboscidea. The Tasmanian devil sequence was removed from the original phylogenetic analysis, as it could not be placed within the tree with confidence. This was not unexpected as marsupials are estimated to have diverged from placental mammals between 185–225 million years ago [53].

### Comparative Cross Species Analysis of Intron 16

Conservation across the orthologous horse, dog, rabbit, elephant, human, mouse and Tasmanian devil *ACE* genes is illustrated in Figures S2 and 3. Introns 12, 16 and 20 had conservation levels nearing that of the exons. Intron 12 is known to contain the testicular ACE promoter, and as such a high level of sequence similarity is expected in this area. The conservation across intron 20 is not apparent in the rodent or marsupial, and no further analysis was carried out in this region. Further conservation analysis using rankVISTA across the entire gene confirmed that intron 12 and 16 were the only non-coding regions to show significant conservation across all species examined except the marsupial, so intron 16 became the focus of further analysis.

**Figure 3:**
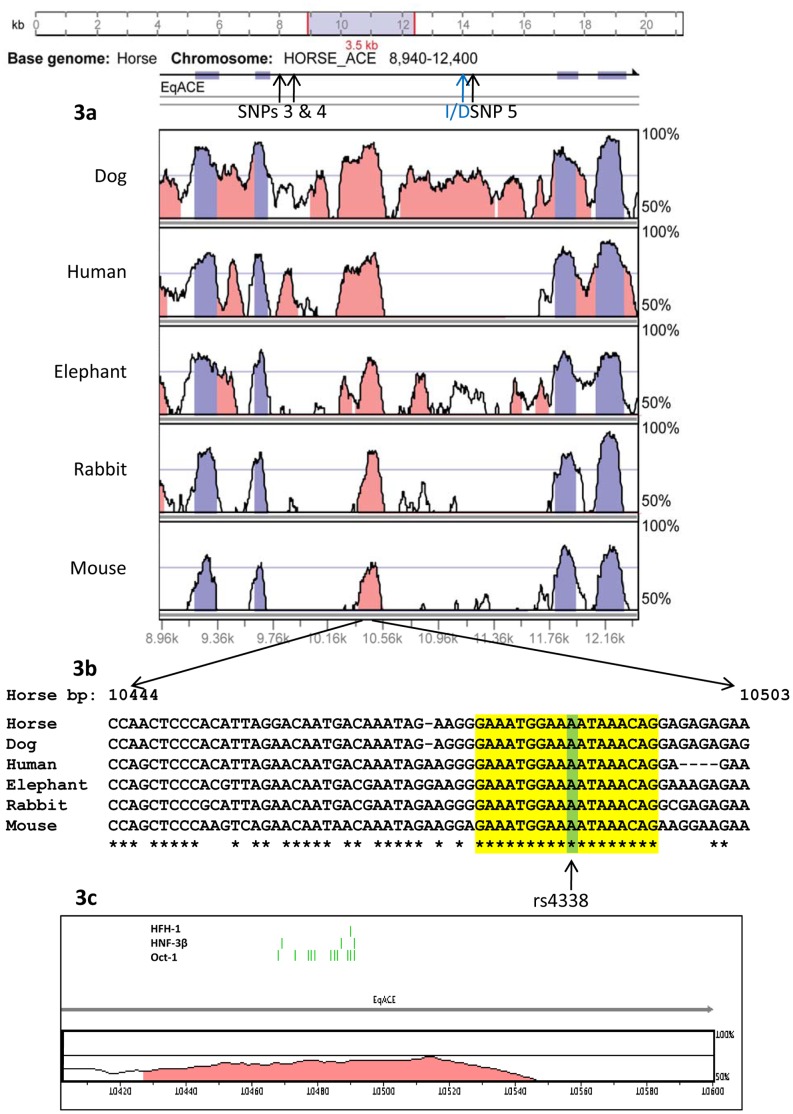
Sequence conservation across intron 16 of multi species *ACE* genes. (a) Plot of conservation spanning exons 15 to 18 between the developed equine *ACE* sequence and the reference sequences from the dog, human, elephant, rabbit and mouse. Pink coloured regions are >70% conserved between the horse and query sequence; while the dark blue regions are annotated exons. Pink conserved peaks are clearly visible in intron 16. The positions of common equine SNPs are marked with black arrows, and the human I/D variant is noted in blue. The human intron sequence analysed here was of the deletion allele. (b) Aligned intron sequence from six species showing the conserved nucleotides highlighted in yellow. The position of SNP rs4338 that occurs in the middle of the human CNE is highlighted in green. (c) Results of the rVISTA scan showing likely motif binding sites (green bars) for the three identified transcription factors.

Figure 3a shows the conservation plot between the horse and the dog, human, elephant, rabbit and mouse intron 16 sequences. These species were included as representative species of the more diverse major clades of placental mammals (Perissidactyla, Carnivora, Primates, Proboscidea, Lagomorpha, and Rodentia; Figure 2). The marsupial was dropped from the analysis because it did not show conservation in this region. Within this intron lay a 380 bp region that was 77% identical between the horse and human (calculated in rankVISTA), while random genomic sequences are expected to be around 33% identical [42]. Within this conserved sequence, an 18 bp sequence was found to be identical across the six species investigated (Figure 3b).

Since this small sequence has been conserved over a range of placental mammals that are evolutionarily separated by around 103 million years [53], it is possibly a conserved nuclear element (CNE). Its absence from the marsupial places its origin between 103–185 million years ago. The conserved nuclear element could potentially be a *cis*-regulatory element. These elements are known to have similar or even lower conservation than promoter regions but still show a significant level of conservation due to selection pressure to maintain their activity, by elimination of mutations that disrupt function [54]. More divergence in these sequences (compared to protein coding and promoter sequences) is tolerated as there is some flexibility in the binding sites within *cis*-regulatory elements [54].

Comparative genomics has identified many non-protein-coding conserved sites in mammalian sequences by cross species comparison. Although few of these sites have defined function, it is thought they play important biological roles [55]. A study by Shen *et. al.* found that most *cis*-regulatory elements identified in the mouse genome functioned by modifying transcription [55], and this effect is often tissue specific. Comparison of the homology of 320 kB genomic DNA surrounding the human, mouse and chicken stem cell leukaemia genes was successfully used to identify known and new enhancer elements [56]. Similarly, a novel sequence was found to regulate the interleukin-4, -5 and -13 genes across a number of mammalian species by comparing the sequence surrounding these genes [57]. Other examples are reviewed by Nobrega and Pennacchio [58], who also recognised that similar strategies will probably identify many more gene regulatory elements in the human genome.

A search for transcription factor (TF) binding sites across *ACE* intron 16 identified three TFs that were predicted to bind to the 18 bp CNE region by at least four of the five programs used (ConSite, TFSearch, MAPPER, Alibaba2 and rVISTA). These were octamer-binding protein-1 (Oct-1), and hepatocyte nuclear factors 3-beta (HNF-3β, also known as FoxA2) and homologue-1 (HFH-1, also known as FoxQ1). The Fox family of factors are expressed in a range of tissues and have a wide ranging number of roles, including contributing to embryonic development, cell cycle regulation, cellular signalling and regulation of tissue specific gene expression [59], [60]. In particular, HNF-3β is essential for notochord formation in embryonic development and regulates cell specific transcription in hepatocytes, and respiratory, intestinal, oesophageal, stomach and pancreatic epithelium [61]–[64]. Oct-1 is a ubiquitously expressed transcriptional regulator that is essential for embryonic survival and normal erythropoiesis [65], [66]. Oct-1 is also thought to be a sensor for metabolic stress, recognising cellular stress and modulating gene expression in response [65], [67]. Further functional studies are required to verify whether any of these transcription factors interact with the CNE region in intron 16 of the ACE gene.

We combined comparative sequence analysis with a search of known transcription factor binding sites to underpin the likelihood of identifying a functional non-coding regulatory region [68]. When tested for more than 100 wide ranging functional binding sites, the ConSite service retained around 70–80% of validated sites, whilst eliminating a number of false positives [69]. A similar process has also been used to identify regulatory modules across different species of *Drosophila* [70], [71]. We consider it possible that the 18 bp CNE in intron 16 encodes a transcription factor binding site, although further functional analysis is needed to determine which of the factors might have an effect on ACE expression; in which tissues, and to what extent this modifies expression of the gene.

### Polymorphism and Haplotype Analysis

Over 10 kb of sequence derived from 35 individuals and including 73% of the cDNA was screened for polymorphisms, resulting in the identification of 16 sequence changes (Figure 1, Tables 1 and S1). Eleven single nucleotide polymorphisms (SNPs) were identified in non-coding sequence and four were observed in coding sequence, including three that were silent, and one causing a conservative amino acid change (p.Arg1290His). This polymorphism is predicted to be within the intracellular region of the protein and as such is unlikely to play a role in circulating enzyme function. Additionally, the rat possesses a histidine in this position, so we expect that this exchange has no major effect on gene function. No I/D polymorphism was detected in intron 16, while a variable length poly-A stretch was identified in intron 14 associated with the equine repetitive element.

The use of pooled DNA and targeting the screen at coding regions decreased the number of polymorphisms detected in this study compared to those discovered in a similar study of the human *ACE* gene [72]. However, the proportion of common polymorphisms detected was comparable between the two species (63% of equine SNPs were found in more than one individual, compared to 67% in humans), confirming the utility of pooled DNA for detection of medium frequency common SNPs. An in-depth analysis of variation in the canine *ACE* gene in 100 individual dogs of different breeds identified 81 variants, including 4 in exons [73]. Although our study found fewer intronic variants, we only covered half the gene in our scan, in addition to using pooled DNA. Furthermore, we found 4 variants in coding sequence and another in the 3′UTR of exon 26, which was comparable to the number found in the higher coverage canine scan.

**Figure 4:**
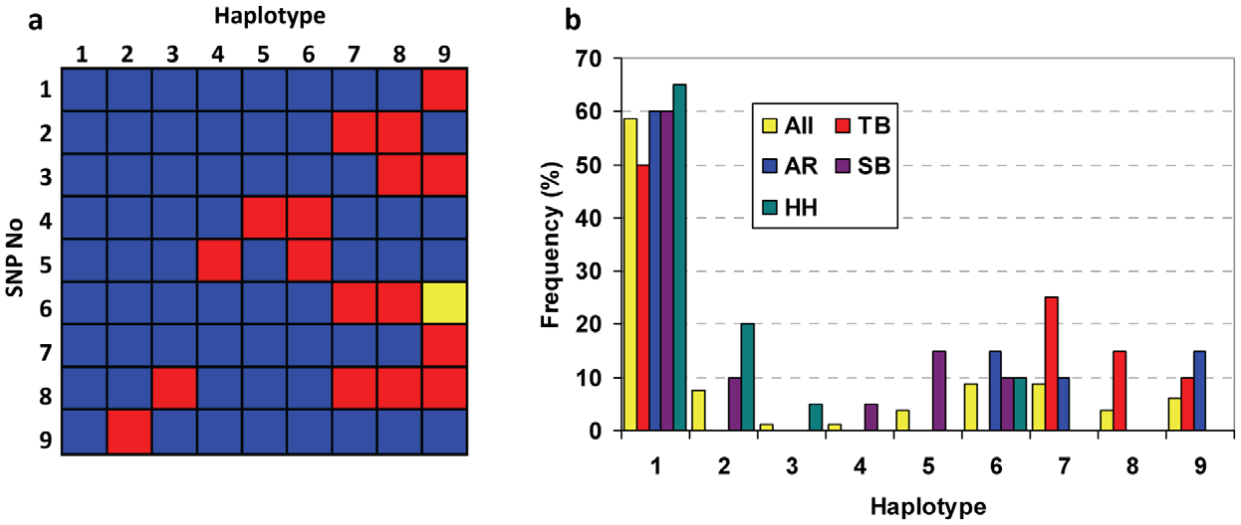
Haplotypes identified in the equine *ACE* gene. (a) Haplotype representation, with blue boxes showing major alleles, red boxes minor alleles and the yellow box shows the third allele of SNP 6. (b) Haplotype distributions across the whole population and divided into the different breeds examined: Thoroughbred (TB), Arabian (AR), Standardbred (SB) and heavy horses (HH, including Clydesdales and Shires).

Nine SNPs were found in more than one animal (allocated SNPs 1–9, Table 1) and were genotyped across the panel of four different horse breeds (Standardbreds, SB; Arabians, AR; Thoroughbreds, TB; and heavy horses, HH). The nine equine SNPs were resolved into nine likely haplotypes (Figure 4a). From the 80 possible representations, one haplotype (H1) was represented 47 times, two (H6 and H7) 7 times, one (H2) 6 times, one (H9) 5 times, two (H5 and H8) 3 times and only two haplotypes (H3 and H4) were unique, in the HH and SB populations, respectively.

The haplotype containing the most common allele of each SNP occurred most frequently in our population. However, with the exception of haplotype 6 (which was seen in all breeds except the Thoroughbred), the other haplotypes appeared to occur either in the light horses (TB and AR, haplotypes 7, 8 and 9) or the heavy horses (SB and HH, haplotypes 2, 3, 4 and 5, Figure 4b). Due to the small numbers of horses screened it is not possible to identify whether the differences in haplotype distribution between breeds is significant and related to selective breeding for differential performance, or due to founder effects and closed studbooks. Haplotypes 4, 5, 8 and 9 contained one polymorphism within intron 16, while haplotype 6 contained two in complete linkage disequilibrium. Although none of these SNPs were within the 17 bp conserved sequence, SNPs 3 and 4 (in haplotypes 5, 6, 8 and 9) were within the first part of the intron that showed high homology to the corresponding human intron.

Although none of the equine SNPs identified coincide with the predicted TF binding site, at least one human SNP is known to occur at base 10 of the 18 bp CNE (Figure 3b). This A to G transition (rs4338) is predicted to reduce affinity for this site and thus prevent binding of all three of the identified transcription factors, potentially altering gene transcription. This SNP is not in complete linkage disequilibrium (LD) with the I/D polymorphism, and is rare in the human population, with a minor allele frequency of approximately 0.017% [74]. The G allele is associated with the deletion *Alu* allele and higher circulating enzyme levels [72].

This SNP in particular warrants specific testing in association studies in humans. Other SNPs in close proximity, rs4334, rs4336 and rs4337 (287, 96 and 94 bp upstream of rs4338 respectively) have been observed to be in complete LD with the I/D polymorphism [72] and occur in the highly conserved region just upstream of the putative binding site. The observed variation in human circulating ACE levels attributed to the I/D polymorphism may actually be due to the action of one of these three SNPs. SNPs 3 and 4 in the horse are also within this conserved region, although further upstream (672 and 583 bp). It is possible that any polymorphism within the conserved region will affect *cis*-regulatory function and any variation in the whole region could contribute to the observed variation in circulating ACE levels.

Previous studies have identified other polymorphisms responsible for variation in circulating ACE. Both an animo acid exchange P1199L and the nonsense *ACE* mutation W1197X dramatically increase circulating ACE levels, with these polymorphisms affecting cleavage of the enzyme from the cell membrane (secretion of the enzyme) and thus circulating ACE levels rather than transcription and membrane bound ACE levels [75], [76]. Other studies have focused on identifying polymorphisms that instead alter gene expression. One variant, rs4343 (G2350A), has been identified as accounting for 19% of the variance in ACE in 1343 Nigerians from 332 families [24]. This SNP also had the strongest association with serum ACE activity in a genome wide association performed on over 1000 individuals [77]. Additionally, this polymorphism was incompletely linked to ACE levels in two other studies, although the association disappeared in one when the analysis was adjusted for the effect of the nearby I/D [22], [23]. The rs4343 polymorphism occurs in exon 17, just downstream of the CNE region; and the incomplete linkage indicated that other polymorphisms also affect circulating ACE levels in the populations examined [22]. These studies did not take into account all known *ACE* polymorphisms, or any polymorphisms from intron 16 in particular, with the exception of the I/D. Further DNA binding assays should be undertaken in both human and horse to determine whether these SNPs affect ACE expression levels through differential binding of a TF, or if one of the other SNPs within intron 16 contribute to variation differences in *ACE* gene expression. Additionally, any further study into the effect of gene variants on circulating enzyme levels needs to account for all known variants in the analysis.

With the development of SNP chips and high throughput (next generation) sequencing, genome-wide approaches are the preferred methods for identification of genetic variants underlying phenotypic traits. This is particularly true for complex traits such as racing performance which have many genes with small effects, in addition to environmental factors, contributing to overall success [78], [79]. Candidate gene analysis such as this study are less powerful compared to whole genome analysis to identify causative genetic variants, although numerous genes expected to affect racing performance have been identified [80]. Notably, a successful candidate gene study was published in 2010, where variation in the equine myostatin (*MSTN)* gene was shown to be significantly associated racing performance in Thoroughbred racehorses [81]. The *MSTN* SNP, which is strongly associated with whether a horse is better suited to sprinting (≤1600 m or 8 furlongs) or staying (>1600 m) races, is located within a putative transcription factor binding site in intron 1 [82]. The SNP is also associated with *MSTN* mRNA changes in response to training; although the mechanism by which this occurs is unknown [83]. Our study was originally performed to investigate the association between racing performance and *ACE* gene polymorphisms in the horse. Further studies are now underway to investigate any association between the polymorphisms identified and racing performance.

### Conclusions

We have performed an extensive study of the sequence and structure of the equine *ACE* gene, and identified common haplotypes of the gene across a diverse cohort of breeds. We identified a conserved non coding element within intron 16 that is shared across representatives of the major placental mammalian lineages. It provides a new focus for the identification of functional variants within the *ACE* gene that affect enzyme levels and biological performance. Soubrier and colleagues [84] noted that since *ACE* has been extensively and systemically sequenced it is likely that all the functional variants have been detected, but their identification is impeded by their almost complete LD with the I/D polymorphism. Further study of the SNPs recognised in this study (both within the horse and human) may uncover the functional variant that has previously eluded researchers.

## Supporting Information

Figure S1 Alignment of the horse, human and mouse *ACE* gene promoter sequences. Figure S1 part a shows the alignment of the somatic *ACE* promoters with the TATAA box highlighted in yellow. Putative SP1 binding sites known to be functional in the human are indicated in green. Part b shows the alignment of the intronic testicular *ACE* promoters. The TTATT sequence is highlighted in yellow and the predicted cAMP-responsive element binding site in green.
(DOCX)

Figure S2 Full multi-species alignment of the *ACE* gene. The alignment shows the conservation between the developed equine ACE sequence with the dog, human, elephant, rabbit, mouse and Tasmanian devil orthologous *ACE* gene sequences [EnSembl: ENSCAFG00000012998, ENST00000290866, ENSLAFG00000006295, ENSOCUT00000001559, ENSMUST00000001963 and ENSSHAT00000012503 respectively]. Regions that are coloured pink are >70% conserved between the reference and query sequences, and the dark blue regions are annotated exons. Exon 13, which is not transcribed into the sACE protein, is not annotated. Pink conserved peaks are clearly visible in introns 12 and 16 (which are labelled) across most species, but not in other introns.
(DOCX)

Table S1 Primers for characterization of the equine *ACE* gene. All primers were used for sequencing of PCR product with the exceptions of Aceex1rev, AceI14rev Aceex26for, which were used for direct BAC sequencing. Primer pairs also used for screening the DNA pools are marked (*).
(DOC)
