# Supplementary material for: Multi-Species Comparative Analysis of the Equine ACE Gene Identifies a Highly Conserved Potential Transcription Factor Binding Site in Intron 16
Source: PLoS One. 2013 Feb 8;8(2):e55434. doi: 10.1371/journal.pone.0055434 (PMC3568152; doi:10.1371/journal.pone.0055434)
Supplement: Figure S1 — Alignment of the horse, human and mouse ACE gene promoter sequences. Figure S1 part a shows the alignment of the somatic ACE promoters with the TATAA box highlighted in yellow. Putative SP1 binding sites known to be functional in the human are indicated in green. Part b shows the alignment of the intronic testicular ACE promoters. The TTATT sequence is highlighted in yellow and the predicted cAMP-responsive element binding site in green. (DOCX) [file pone.0055434.s001.docx]

**Figure S1a**

Horse GCCCTGACCTAAGCCCCATCCGCCACCCCTGGGCTGCATGTCGCAGGGGGACACCTCAGC 60

Human ---CACCCCCGGGCTGCA--------CCTCGGCCCC----TCCCCGG-------CCCGCG 38

Mouse --------CCAGGCTGC--------TCCCTGGCCC------CACAGGG------CTC--- 29

* ** * ** ** * * * ** * *

Horse TCCT--CCCCGGGCGGGCCGGGAAGCGTCGGTCCCCGCCCCGCTCCGTTAGGCCCGCGCT 118

Human CCCCTGCCCGGGGCGGGCCAGGAA----------------------CCTCGGCCCGCGCC 76

Mouse TCCTTGCCCAGGGCGGGCGGGGAA-------------------TCAGCCGGGTTCGCGCT 70

** *** ******** **** ** *****

Horse GCCGGGGGACTTTGCAGCCGAGAAGGAAGCGCGGCGGGGCGGGGAGGTGGGGGTGTGTCG 178

Human GCTGGGG-ACTTTGGAGCGGAGGAGGAAGCGCGGCGGGGCGGGG--GCGGGGGTGTGTCG 133

Mouse GCCGGGG-ACTTTGGAGC-GAGGAGGAAGCGCGGAGGGGCGGGGAGGTGGGGGTGTGTCG 128

** **** ****** *** *** *********** ********* * ************

Horse GAAGGCGGCGGCGGCCGCCGGGTTTTATAATCCGCAGGGCGGACGCGGCGCGGGAGAAGG 238

Human G--------------------GTTTTATAACCCGCAGGGCGGCCGCGGCGCAGGAGAAGG 173

Mouse GGAGGCGGTGGTGGCTG---GGTTTTATAATCCGCAGGGCGGTCGCGGCACGAGAGAAGG 185

* ********* *********** ****** * *******

Horse GGCAGCACCGCGCGCCGCGCACCGCGCCATGGGGG 273

Human GGCAGAGCCGAGCACCGCGCACCGGGTCATGGGGG 208

Mouse GGCAGCGCCGCACCGCGCGCACCGCGCCATGGGGG 220

***** *** * ********* * ********

**Figure S1b**

Horse GTAAAGCCCTGAGGGAGGGTGGGTTGGGGCACTAAGGCGAGTTCTCAATTCTGG------ 54

Human GTAAAGCCCTGAGTGAGGATGGTGTGGGG--CTAAGGTGGGTCCTCAACTCTGG------ 52

Mouse GTAAAGCTCTGAGTGAGGGTGGACTGGGA--CCAAGAGAAGTCCTGGCCTCTGGCCTCTG 58

******* ***** **** *** **** * *** ** ** *****

Horse GCT--------CTGGACCCAGGTTCCCCATCAGCTC-CTCCCAGCTGGGCCCTGGTACCC 105

Human GCTT---GGCCCAGGCCCCAGGTTCCTGGTCAGCTC-CTACCAGCTGAGCCCTGGTACCC 108

Mouse GCTTCTGGGTCAAAGCCTCAGCATCCTGGTCACTTTGCTGCCAGCTGAGCCCCAGTGTCC 118

*** * * *** *** *** * ** ******* **** ** **

Horse TGCCCTGGAGGGCCAGGCAGCCCTCTGAGCTCATCAGCAGGGCCCTGGGGGAGAGACGGG 165

Human TGTCCTGGAGGGCCAGGCAGCCCCCCAAGCTCATCAGCAGGGCCTGCGAGTGGGGACAGG 168

Mouse TTTGCTTCAGTGCCAAGCCACCC-CTGGGCTCATCCTCAGGGCCCT-AAGCAGAAATGGG 176

* ** ** **** ** *** * ******* ******* * * * **

Horse AATGTCTTTTCCCCAGCGTCCTAGAGAGGGTATGCCCAGATCTGAGGGCCCC-------- 217

Human CATGTCTTTCCCCCAGCATCCTAGAGAGGGTGTGCTCAGACCTGAGGGCCCCTCCCCTTC 228

Mouse TATGTCTTTCTCTCAGGGTCCTAGAGACAGTGTGCCCAAGCCTGAGGGCCCTT------- 229

******** * *** ********* ** *** ** **********

Horse -AGGGACAGGCTGTCTGGCCACACTGCTTTATGAGGTCACACTGCAGGCTCCTCTTTTAT 276

Human CAGAGGAAG-----CCAGACACAAGGCTCTGTGAGGTCACACTGCGGGCTCCGCTCTTAT 283

Mouse -GGGGTCAGGCTGGCTGG-CACATTGCTCTATGAGGTCACACTGCAGGCTTGGCTCTTAT 287

* * ** * * **** *** * ************** **** ** ****

Horse TGGCCAGGGTGTGGT 291

Human TGGCCAGGGGACGGT 298

Mouse TGGCC-GGTGATGGG 301

***** ** **
