# Supplementary figures and images for: Multi-Species Comparative Analysis of the Equine ACE Gene Identifies a Highly Conserved Potential Transcription Factor Binding Site in Intron 16
Source: PLoS One. 2013 Feb 8;8(2):e55434. doi: 10.1371/journal.pone.0055434 (PMC3568152; doi:10.1371/journal.pone.0055434)

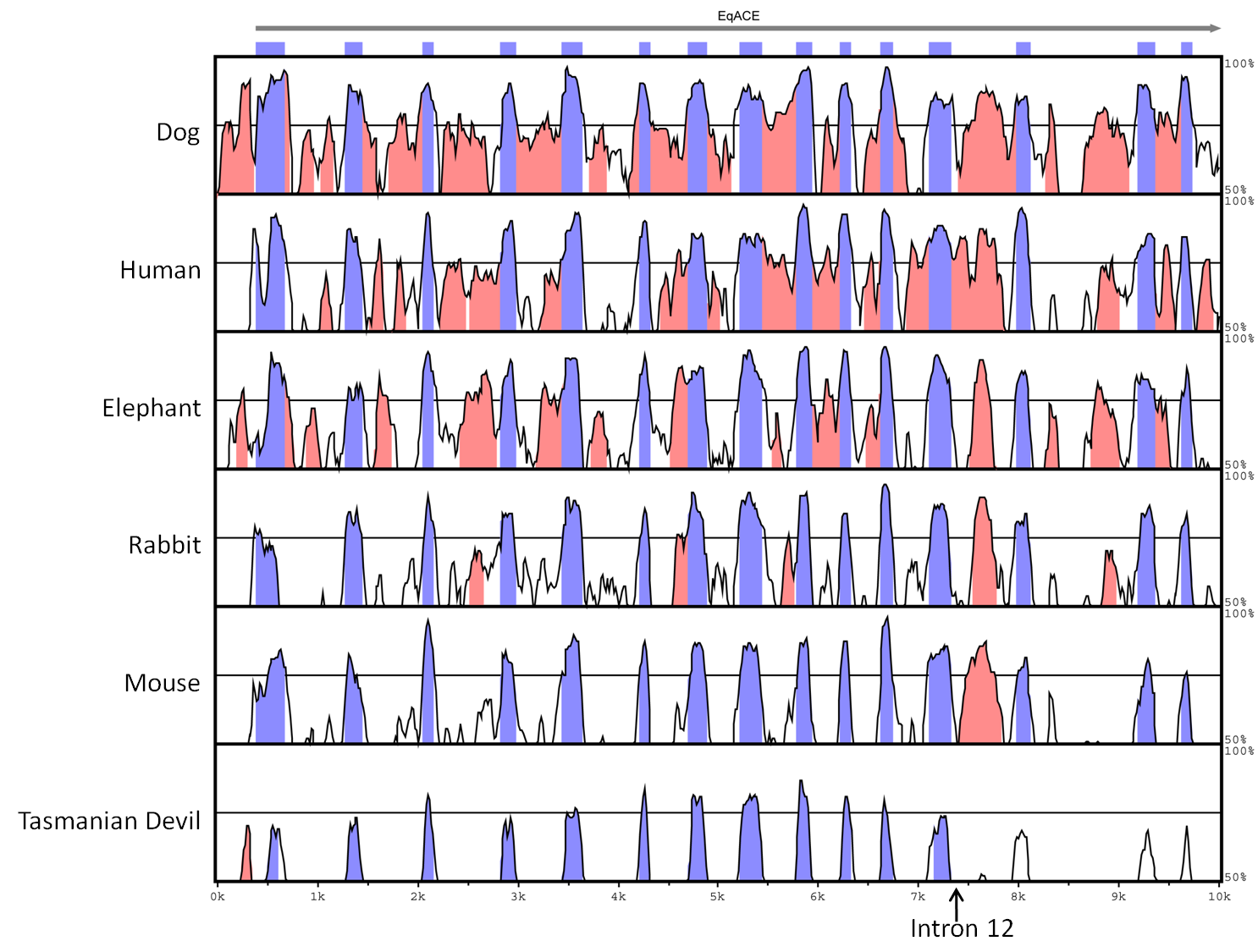


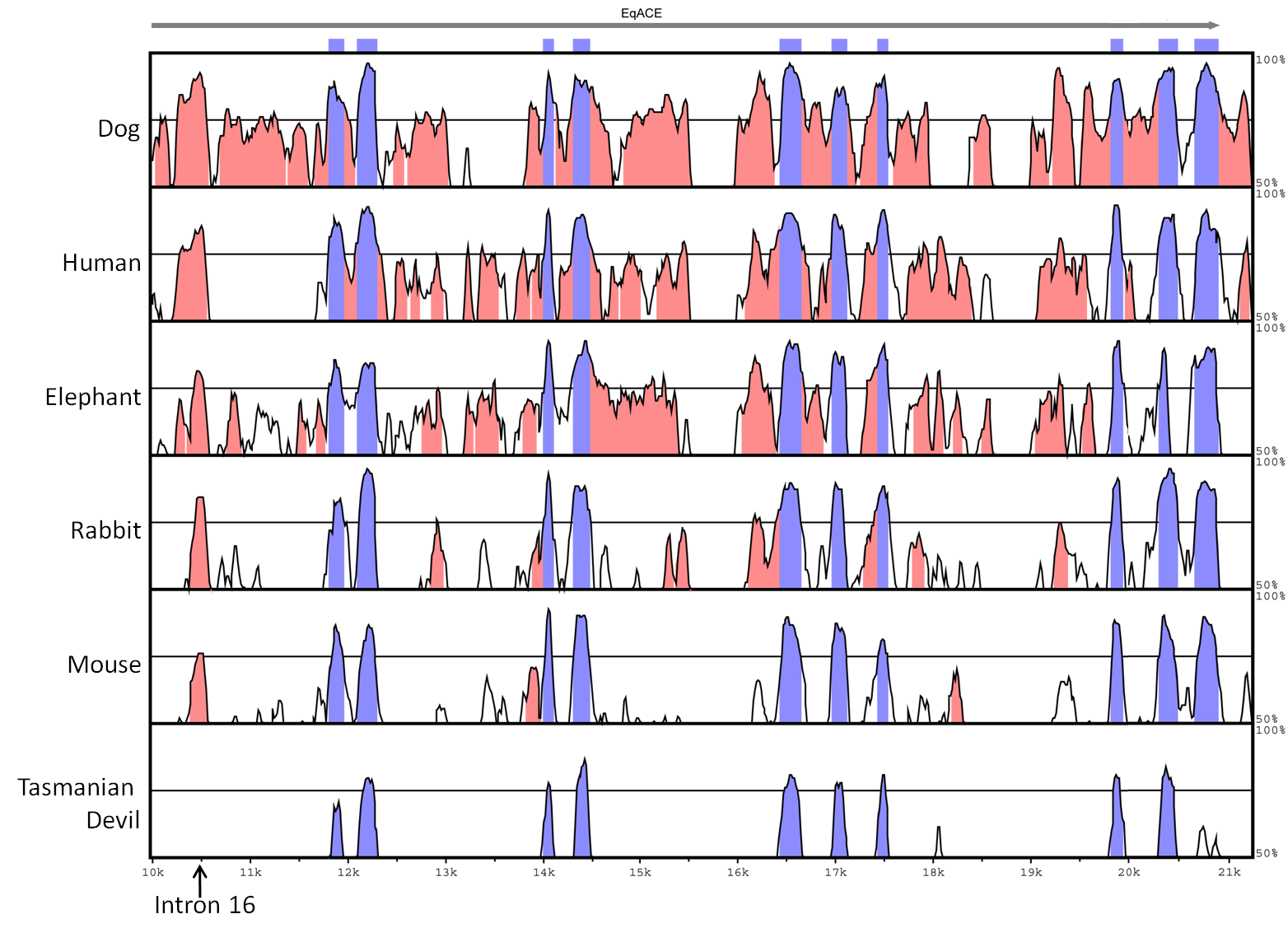

Supplement: Figure S2 — Full multi-species alignment of the ACE gene. The alignment shows the conservation between the developed equine ACE sequence with the dog, human, elephant, rabbit, mouse and Tasmanian devil orthologous ACE gene sequences [EnSembl: ENSCAFG00000012998, ENST00000290866, ENSLAFG00000006295, ENSOCUT00000001559, ENSMUST00000001963 and ENSSHAT00000012503 respectively]. Regions that are coloured pink are >70% conserved between the reference and query sequences, and the dark blue regions are annotated exons. Exon 13, which is not transcribed into the sACE protein, is not annotated. Pink conserved peaks are clearly visible in introns 12 and 16 (which are labelled) across most species, but not in other introns. (DOCX) [file pone.0055434.s002.docx]
