# Supplementary material for: Multi-Species Comparative Analysis of the Equine ACE Gene Identifies a Highly Conserved Potential Transcription Factor Binding Site in Intron 16
Source: PLoS One. 2013 Feb 8;8(2):e55434. doi: 10.1371/journal.pone.0055434 (PMC3568152; doi:10.1371/journal.pone.0055434)
Supplement: Table S1 — Primers for characterization of the equine ACE gene. All primers were used for sequencing of PCR product with the exceptions of Aceex1rev, AceI14rev Aceex26for, which were used for direct BAC sequencing. Primer pairs also used for screening the DNA pools are marked (*). (DOC) [file pone.0055434.s003.doc]

**Table S1. Primers for characterization of the equine *ACE*** gene.

| **Area of Gene** | **Primer Name** | **Sequence 5’ to 3’** | **Product size (bp)** | **Comments** |
| --- | --- | --- | --- | --- |
| 5’ UTR- Ex 1 | Aceex1rev | TGGGTGATGTTGGTGACG | 624 | Direct sequencing of BAC used |
| Ex 1 – Int 1 | Ace1s.7 | CTGCTGCCACCGCCGC | 438 |  |
|  | Ace1int.2 | GAGCCAGCCTCATCCTTCG |  |  |
| Ex 1 – Int 2 | Ace1.3 | CGCACGACACCAACATCAC | 945 |  |
|  | Ace2int.2 | CAGTCAGGACCCCTACAGA |  |  |
| Ex 2 – Ex 3 | Ace2.1 | GAGGAAGMRGCCCTGMTCA | 858 |  |
|  | Ace3.2 | GCAGGTGGCAGTCTTGTT |  |  |
| Int 2 – Ex 4 | Ace2int.1 | TTCATCGCTAACATTTTCTCG | 887 |  |
|  | Ace4.2 | GTTGTGCCAGCCCTCCCA |  |  |
| Int 3 – Ex 5 | Ace3int.1 | GCTCTGGTGAAGGCTGTTA | 851 |  |
|  | Ace5.2 | TGGGTCCCCTGAGGTTGAT |  |  |
| Ex 5 – Int 5* | Ace5.1 | CCTACTGGCGCTCCTGGTA | 585 | SNP1: c.868+25A>G |
|  | Ace5sint.2 | GAGTGCTCTGTGTTCCCGA |  |  |
| Int 5 – Int 6* | Ace5sint.1 | TCGGGAACACAGAGCACT | 494 |  |
|  | Ace6sint.2 | CACCAGGGGGTCCTAAAG |  |  |
| Ex 6 – Ex 7 | Ace6.1 | CGATGTCACCAGCACTATG | 600 |  |
|  | Ace7.2 | CTGAAGTCTTTTCTGTTGTAGAAGTCC |  |  |
| Ex 7 – Ex 8 | Ace7.1 | AATGCTACGCACATGTTCT | 725 | Singleton SNP in exon 8 in BAC: c.1155G>A |
|  | Ace8.2 | TGTCATTGGTGACACGGTCCAG |  |  |
| Ex 8 – Ex 9* | Ace8.5 | GTCTCCACCCCTACACA | 550 | SNP2: c.1363+146G>T |
|  | Ace9.2 | CGAAGATACCACCAGTC |  |  |
| Ex 9 – Int 10* | Ace9s.1 | GACAGAAAGCGACATCAAT | 604 |  |
|  | Ace10sint.2 | GCAGGGGACTAAAGGTG |  |  |
| Int 9 – Ex 11 | Ace9int.1 | GCTAAATCAGCCTGTGTGC | 562 |  |
|  | Ace11.2 | GCCCCTGCCTTGGTGGAC |  |  |
| Int 10 – Int 11* | Ace10sint.1 | CTGGTTGGGCTTCTGTCC | 543 | Singleton SNP in intron 11 in BAC: c.1731–109C>G |
|  | Ace11sint.2 | GTCTCAGGCTGGAGTTCAC |  |  |
| Ex 11 – Ex 12 | Ace11.1 | GCACCAGTGTGACATCTA | 622 |  |
|  | Ace12.2 | ATGCTCTCTGGGTAATTGT |  |  |
| Ex 12 – Ex 13* | Ace12s.5 | GCAAGAGGTGCTGAAGGA | 636 |  |
|  | Ace13s.4 | TGCCTGGCTGGTTGTTC |  |  |
| Ex 13 – Ex 14 | Ace13.1 | CTGCCCAGCCTCCTCTTC | 370 |  |
|  | Ace14.2 | CGGTCATACTCCTCCACGA |  |  |
| Ex 13 – Int 14* | Ace13s.3 | TCAACCAGGGAACAACCAGC | 509 |  |
|  | Ace14sint.6 | ACCCCCAAGGGAAGGAGG |  |  |
| Int 14* | AceI14for | TGCTGTGGTAGGCGTCCC | 218 | Variable length repeat in all breeds c.2079+508-512A(12_16) |
|  | AceI14rev | AGTTCTCTGTGGCTCCTCTTGA |  |  |
| Int 14 – Ex 15 | Ace14int.1 | TCTTTCCCTCCTTCCCTT | 1093 |  |
|  | Ace15.2 | GTCCTGAACCTTCTTTATGA |  |  |
| Int 14 | AceI14rev | AGTTCTCTGTGGCTCCTCTTGA |  | Direct sequencing of BAC used |
|  |  |  |  |  |
| Int 14 – Int 15 | Ace14int.3 | CAGGCAAAGACGGCAACT | 566 |  |
|  | Ace15int.2 | CAAGAGGACGGTTCAGAGGC |  |  |
| Int 15 – Int 16* | Ace15int.5 | GCCTGCTGCCTCTCTTCTT | 396 | Intron 16 SNP 3: c.2326+89C>G; singleton in HH: c.2326+99C>T;  SNP 4: c.2326+178G>A. |
|  | Ace16sint.14 | AGGGTATGGCACAGGGAG |  |  |
| Ex 16 – Int 16 | Ace16.1 | ATGGAGACCACTTACAGCGT | 830 |  |
|  | Ace16int.2 | TTCCCTTCTATTTGTCATTGT |  |  |
| Int 16* | Ace16sint.5 | CATCTGCTCCCTCTCCGT | 547 |  |
|  | Ace16int.2 | TTCCCTTCTATTTGTCATTGT |  |  |
| Int 16* | Ace16sint.7 | GCCCAACTCCCACATTAG | 472 |  |
|  | Ace16sint.6 | GCTCACTCTCCTTATTTCGG |  |  |
| Int 16* | Ace16int.3 | CCGAAATAAGGAGAGTGAG | 509 | SNP 5: c.2326-583G>T |
|  | Ace16sint.8 | CCCACTGACACCAAAATC |  |  |
| Int 16* | Ace16sint.13 | GCTCCTGTTCAATCTTCACC | 486 |  |
|  | Ace16sint.10 | AGCCCTTCGCTCACCTC |  |  |
| Int 16 – Int 17* | Ace16sint.15 | CCCACCCTTTCTCCTATT | 223 |  |
|  | Ace17int.2 | AGAGCCAGTGATGCCAG |  |  |
| Ex 17 – Ex 18* | Ace17.1 | GCTGGCGAGACAAGGTGG | 423 | Singleton SNP in exon 18 in BAC: c.2484G>T |
|  | Ace18.2 | AGCAGGTGAGCAGGAATGGG |  |  |
| Ex 18 – Ex 19 | Ace18.3 | CCGCTCTACCTGAACCTGC | 1772 | Gap in sequence |
|  | Ace19.2 | CGTGGGGGCTGAAGGGAA |  |  |
| Int 18 – Ex 19 | Ace18int.1 | ATAGGAGCGTGAGGAAGGGG | 1329 | Gap in sequence |
|  | Ace19.2 | CGTGGGGGCTGAAGGGAA |  |  |
| Ex 18 – Ex 20* | Ace18sint.3 | TGGTTCGCCTCACCCTGT | 533 |  |
|  | Ace20.2 | CTTGCCGTTGTAGAAGTCCCA |  |  |
| Ex 20 – Int 20* | Ace20s.3 | AAGCCAACTGATGGACGG | 502 | Intron 20 SNP 6: c.2933+58G>A>C;  SNP 7: c.2933+115G>T |
|  | Ace20sint.4 | CTGAGCCTCTGTTACTGGTGA |  |  |
| Int 20 – Int 20 | Ace20int.1 | GCTTGCCCATTGGATTCT | 1339 |  |
|  | Ace20int.2 | GGTAGGGAGAGGGTGTTGA |  |  |
| Int 20 – Int 21* | Ace20int.3 | GCAGTAAGGACAGCAGTT | 431 | Intron 21 SNP 8: c.3157+39C>A |
|  | Ace21int.2 | CCCCATTATTCACCATTG |  |  |
| Int 21 – Int 22* | Ace21sint.1 | GGGATAAAGAAGGGGCAG | 487 |  |
|  | Ace22sint.4 | GCACACTCACACAGACACC |  |  |
| Ex 22 – Ex 23 | Ace22.1 | AGCAYGACATCAACTTYCT | 542 |  |
|  | Ace23.2 | GCCCCTGGGTCAAAGTCA |  |  |
| Int 22 – Int 23* | Ace22sint.3 | ACAGAGGCACAGCACGCA | 423 |  |
|  | Ace23sint.4 | TGTCAAAAGAGTGAAGCAATGG |  |  |
| Ex 23 – Ex 24 | Ace23.1 | AAGGTGACTTTGACCCAGG | 2202 |  |
|  | Ace24.2 | CGTGGAACTGGAACTGGA |  |  |
| Int 23 – Int 23 | Ace23int.1 | AGCCTCAGTTTCCTCACCT | 1431 |  |
|  | Ace23int.2 | GTCCCTTCCACGCCTCC |  |  |
| Int 23 – Int 24* | Ace23sint.5 | CTCCAACCACCCCACTCTC | 432 |  |
|  | Ace24sint.2 | CGCTCTACCAGCCTGAACTT |  |  |
| Int 24 – Int 25* | Ace24sint.3 | ATGTGCCATCTCCAGTG | 503 |  |
|  | Ace25int.2 | GCTTCCCTCTCCTTGCTC |  |  |
| Int 25 – Int 26* | Ace25int.1 | CTCCCCAGTTCAGGCAT | 396 | Exon 26 singleton SNP in QH: c.3813C>T; SNP 9: p.Arg1290His;  3’ UTR singleton in HH: c.*163G>A |
|  | Ace26int.2 | GTGTTCCTGTCCCTGTCC |  |  |
| Ex 26 – 3’ UTR | Aceex26for | CCTGGGCTTGAACCTGGAG |  | Direct sequencing of BAC used |

All primers were used for sequencing of PCR product with the exceptions of Aceex1rev, AceI14rev Aceex26for, which were used for direct BAC sequencing. Primer pairs also used for screening the DNA pools are marked (*).
